# Sulforaphane inhibits the growth of prostate cancer by regulating the microRNA-3919/DJ-1 axis

**DOI:** 10.3389/fonc.2024.1361152

**Published:** 2024-03-07

**Authors:** Fangxi Zhang, Xiaofeng Wan, Jianmin Zhan, Ming Shen, Runsheng Li

**Affiliations:** ^1^ National Health Commission (NHC) Key Lab of Reproduction Regulation (Shanghai Institute for Biomedical and Pharmaceutical Technologies), School of Pharmacy, Fudan University, Shanghai, China; ^2^ Department of Pharmacy and Examination, Heze Medical Collge, Heze, China

**Keywords:** sulforaphane, prostate cancer, microRNA-3919, DJ-1, signal pathway

## Abstract

**Background:**

Prostate cancer (PCa) is the second most common solid cancer among men worldwide and the fifth leading cause of cancer-related deaths in men. Sulforaphane (SFN), an isothiocyanate compound, has been shown to exert inhibitory effects on a variety of cancers. However, the biological function of SFN in PCa has not been fully elucidated. The objective of this study was conducted to further investigate the possible underlying mechanism of SFN in PCa using *in vitro* cell culture and in vivo tumor model experiments.

**Methods:**

Cell viability, migration, invasion, and apoptosis were analyzed by Cell Counting Kit-8 (CCK-8), wound healing assay, transwell assay, or flow cytometry. Expression of microRNA (miR)-3919 was detected by quantitative real-time polymerase chain reaction (qRT-PCR) or in situ hybridization assay. Xenograft assay was conducted to validated the antitumor effect of miR-3919. The targeting relationship between miR-3919 and DJ-1 was verified by dual-luciferase reporter assay. The level of DJ-1was measured by qRT-PCR or western blotting (WB).

**Results:**

In the present study, SFN downregulated mRNA and protein expression of DJ-1, an oncogenic gene. Small RNA sequencing analysis and dual-luciferase reporter assay confirmed that microRNA (miR)-3919 directly targeted DJ-1 to inhibition its expression. Furthermore, miR-3919 overexpression impeded viability, migration, and invasion and promoted apoptosis of PCa cells. Tumor growth in nude mice was also inhibited by miR-3919 overexpression, and miR-3919 expression in PCa tissues was lower than that in peritumoral tissues in an *in situ* hybridization assay. Transfection with miR-3919 inhibitors partially reversed the effects of SFN on cell viability, migration, invasion, and apoptosis.

**Conclusion:**

Overall, the miR-3919/DJ-1 axis may be involved in the effects of SFN on the malignant biological behavior of PCa cells, which might be a new therapeutic target in PCa.

## 1 Introduction

Prostate cancer (PCa) is the second most common solid cancer among men worldwide and the fifth leading cause of cancer-related deaths in men (1, 2). Between 1990 and 2010, the number of deaths due to PCa increased from 150,000 to 250,000 worldwide. In 2018, 1,276,106 men were diagnosed with PCa and 358,989 patients died (2). The early stage of PCa mainly depends on the androgen receptor (AR); therefore, androgen deprivation therapy has a good effect. However, most cases relapse and develop castration-resistant prostate cancer (CPRC) (3). Currently, the drugs used to treat CRPC mainly include abiraterone, enzalutamide, docetaxel, cabazitaxel, and sipuleucel-T, but none can markedly improve the survival rate of patients (4). Therefore, there is an urgent need to develop novel therapeutic strategies for CPRC.

Epidemiological studies have shown that the addition of cruciferous vegetables to daily diets plays an important role in the prevention of various tumors, including prostate cancer (5). Moreover, complementary, and alternative medicine exerts pleiotropic effects and lowers the risk of cancer recurrence with fewer side effects (5, 6). Sulforaphane (SFN), a type of isothiocyanate compound, is abundant in cruciferous vegetables and is a natural compound with strong anti-cancer activity (7). A multitude of *in vitro* and *in vivo* experiments have shown that SFN possesses the ability to defend against the occurrence of chemical or radiation-induced carcinogenesis and suppress the proliferation, migration, and invasion of tumor cells (8–10). The antitumor mechanisms of SFN involve various cellular and molecular pathways, including modulation of cell signaling pathways by altering the redox status, suppression of TLR4-mediated transcription, and inhibition of histone deacetylase activity (11, 12). Previous research has demonstrated that sulforaphane exerts its antitumor effects *via* epigenetic modification of the Nrf2 gene, with subsequent activation of the downstream anti-oxidative stress pathway (13, 14). Sulforaphane also can induce autophagy by inhibiting of HDAC6 mediated PTEN activation in triple negative breast cancer cells (15). Another potential mechanism by which sulforaphane interacts with cancers is the regulation of miRNA expression.

MicroRNAs (miRNAs) are a type of noncoding RNAs with approximately 20–24 nucleotides in length that induce the target gene’s mRNA degradation, or inhibit translation initiation and protein synthesis at the post-transcriptional level (16). miRNAs are involved in the occurrence, development, and metastasis of multiple types of human cancer (17, 18). Insights into the functions of miRNAs in tumors have made them attractive tools and targets for new therapeutic strategies (19). For example, miR-181-3p promotes Snail-induced epithelial–mesenchymal transition (EMT) by directly regulating YWHAG, indicating that miR-181-3p may be a novel potential target in metastatic cancers (20). In addition, miR-7 inhibited glycolysis in PCa cells and reshaped the acidic tumor microenvironment, and the sensitization effects of miR-7 overexpression were independent of p53 status, suggesting that miR-7 based agents could be developed for PCa patients, regardless of p53 status (21). Therefore, it is important and helpful for targeted therapy of PCa to investigate the effects of sulforaphane on miRNA expression and to determine the molecular mechanism of miRNAs associated with PCa cells.

Our results demonstrate that sulforaphane regulates the expression of a multitude of miRNAs in PC-3 cells. Furthermore, the miR-3919/DJ-1 axis may be involved in the antitumor effects of SFN in PCa, which may lead to a new therapeutic strategy for PCa.

## 2 Materials and methods

### 2.1 Cell lines and treatment

Human PCa cell lines PC-3 and DU145 were purchased from the National Collection of Authenticated Cell Cultures at the Chinese Academy of Sciences (Shanghai, China). The cells were cultured in Ham’s F-12K (Kaighn’s) medium and MEM medium (Gibco), respectively, supplemented with 10% fetal bovine serum (Gibco).

To confirm the effects of sulforaphane (LKT Labs, St. Paul, MN, USA) on PCa cells, PC-3, and DU145 cells were cultured in their respective media containing different sulforaphane concentrations (0, 5, 10, or 20 μM) for 48 h. For functional experiments, PC-3 and DU145 cells were treated with 10 μM sulforaphane for 48 h. miR-3919 mimics and inhibitors were purchased from RiboBio (Guangzhou, China). The cells were seeded into culture plates and transfected with 50 nM of miRNA mimic/NC or inhibitor/NC using Lipofectamine 2000 (Invitrogen, Carlsbad, CA, USA) at 37 °C for 6 h according to the manufacturer’s protocol. After transfection for 48 h, subsequent assays were performed.

### 2.2 Cell viability assays

The Cell Counting Kit-8 (CCK-8) (Beyotime Biotechnology, China) was used to test cell viability according to the manufacturer’s protocol. Briefly, PC-3 and DU145 cells were seeded in 96-well plates and grown for 24 h. After treatment, the CCK-8 solution was added to each well of a 96-well plate and cultured at 37 °C for 4 h. The optical density (OD) at 450 nm was measured using a Microplate Reader (Infinite M200, Tecan Group Ltd., Männedorf, Switzerland). The relative proliferation rates were calculated.

### 2.3 Wound healing assay

This assay was performed to evaluate the migration of PCa cells. PC-3 and DU145 cells were seeded in 6-well plates (∼70%–80% confluency). After the cells were grown to 100% confluence, they were scratched at the center with a sterile 200-μL pipette tip. The cell debris was washed with PBS. The cells were cultured in serum-free medium. At 0 h and 48 h, the wound area was observed using a light microscope (Olympus, Tokyo, Japan), and the wound healing rate was calculated.

### 2.4 Transwell assays

This assay was conducted to evaluate PCa cell invasion. PC-3 and DU145 cells were trypsinized and resuspended in a serum-free medium. Then, 4 × 10^5^ cells were added to the upper chamber pre-coated with matrix gel in 24-well plates. A culture medium containing 10% FBS was added to the lower chamber. The cells were incubated at 37 °C for 48 h. Subsequently, the cells in the upper chamber were carefully removed. Cells on the other surface of the upper chamber were fixed with 4% paraformaldehyde at 37 °C for 15 min and dried. The cells were then stained with 0.5% crystal violet at room temperature for 5 min. Images of cells were captured using a light microscope (Olympus, Tokyo, Japan).

### 2.5 Cell apoptosis assays

Annexin V-FITC/propidium iodide (PI) double staining was performed to analyze the apoptosis of PCa cells. A total of 3 × 10^6^ cells from each group were collected and resuspended in 100 μL binding buffer, according to the protocol of the Apoptosis Detection Kit (Solarbio, China). Subsequently, 5 μL of Annexin V-FITC and 5 μL of PI were added successively and mixed thoroughly. The reaction was performed at room temperature in the dark for 15 min. The samples were analyzed using a flow cytometer (BD, Germany) within 1 h.

### 2.6 Western blotting

Total protein was extracted from the cells or tissues using RIPA lysis buffer (Beyotime Biotechnology, China). Protein samples (30 μg/well) were separated using 10% SDS-PAGE and transferred onto polyvinylidene fluoride membranes (Millipore, Billerica, MA, USA). The membranes were blocked in 5% non-fat dry milk in TBST for 1.5 h and incubated with specific primary antibodies at 4 °C overnight. Subsequently, the membranes were incubated with an HRP-conjugated secondary antibody at room temperature for 1 h. The protein bands were captured using an ECL system (Tanon 5200, Bio-Tanon, China), and grayscale statistics were performed using Image J software (Bio-Rad, USA). GAPDH was used as a loading control.

### 2.7 RNA isolation and quantitative real-time PCR

TRIzol (Life Technologies, Carlsbad, CA, USA) was used to extract total RNA from tissues or cells. For mRNA, cDNA was obtained using HiScript Q RT SuperMix for qPCR (Vazyme Biotech, Nanjing, China) and RT-qPCR detection was conducted using ChamQ Universal SYBR qPCR Master Mix (Vazyme Biotech, Nanjing, China). DJ-1 expression was normalized to that of GAPDH. For miRNA, cDNA was reversed by miRNA 1st Strand cDNA Synthesis Kit (by stem-loop) and RT-qPCR detection was performed using miRNA Universal SYBR qPCR Master Mix (Vazyme Biotech, Nanjing, China) on a MyiQ Single-Color Real-Time PCR Detection System (Roche, Mannheim, Germany). The expression of miR-3919 was normalized to that of U6. Results were calculated using the 2^−ΔΔCt^ method. The primer sequences are listed in **Table 1**.

**Table 1:** Primers sequences.

GENE	Forward	Reverse
hsa-miR-3919	CGCGGCAGAGAACAAAGGA	AGTGCAGGGTCCGAGGTATT
U6	CTCGCTTCGGCAGCACA	AACGCTTCACGAATTTGCGT
DJ-1	GTCCTACTGCTCTGTTGGCTCA	CCACACGATTCTCAGAGTAGGTG
GAPDH	GTCTCCTCTGACTTCAACAGCG	ACCACCCTGTTGCTGTAGCCAA

### 2.8 Dual-luciferase reporter assay

A dual-luciferase reporter assay was performed to evaluate binding between miRNA-3919 and DJ-1. For luciferase reporter construction, wild-type (WT) or mutant-type (Mut) sequences of DJ-1 3’-UTR were synthesized and inserted into the psiCHECK-2 vector. PC-3 cells were co-transfected with a luciferase reporter plasmid (100 ng) and mimic NC control or miR-3919 mimic (25, 50 or 75 nM) using Lipofectamine 2000 (Invitrogen, Carlsbad, CA, USA). Luciferase activity in the cell lysates was measured using a luciferase reporter assay kit (Beyotime Biotechnology, China) in a Microplate Reader (Infinite M200, Tecan Group Ltd., Männedorf, Switzerland).

### 2.9 Small RNA sequencing

Total RNA was extracted using TRIzol reagent (Life Technologies, Carlsbad, CA, USA) following the manufacturer’s instructions. The quality and quantity of isolated RNA were measured using a NanoDrop spectrophotometer (Thermo Fisher Scientific, USA) and an Agilent bioanalyzer system (Agilent, CA, USA). Sequencing libraries of small RNA were prepared using the NEB Next Multiplex Small RNA Library Prep Set for Illumina (NEB, USA) and sequenced on an Illumina NovaSep 6000 platform (Illumina, USA). The raw reads were filtered to remove adapter sequences and low-quality reads. The remaining reads were used to detect known and novel miRNAs using miRBase. The expression levels of miRNAs were estimated by transcript per million (TPM), and DESeq2 (v1.30.0) was used to analyze differentially expressed miRNAs. *P*-values <0.05, and |log2FoldChange|>1 were considered as standards for differentially expressed miRNAs. The miRNA–mRNA interactions were predicted using miRanda.

### 2.10 Xenograft tumor in mice

The experiment was approved by the Institutional Ethics Committee of the Shanghai Institute of Planned Parenthood Research Center (now Shanghai Institute for Biomedical and Pharmaceutical Technologies) for the commencement of the study. Twelve male BALB/c nude mice (6-week-old) were randomized into two groups based on body weight (six mice per group): an NC group and an miR-3919 agomir group. A single-cell suspension (100 μL) of 5 × 10^6^ PC-3 cells was subcutaneously implanted into the flanks of each mouse. All mice were housed under controlled photoperiod conditions (temperature: 22°C–24°C, humidity: 40%–60%, 12 h light/dark) and were supplied with food and sterilized water *ad libitum*. Tumor volume was measured twice a week and calculated as length (maximum diameter) × width^2^ (vertical diameter) × 0.5. When the tumor volume reached 100 mm^3^, the mice received NC agomir or miR-3919 agomir (5 nmol/mouse) by tumor injection twice weekly. When the tumor volume reached 1,000 mm^3^, the mice were sacrificed under anesthesia, and the tumors were removed and weighed.

### 2.11 miRNA *in situ* hybridization

A tissue microarray, containing 40 paired tumor and adjacent normal tissues was constructed by Shanghai Wellbio Technology Co., Ltd. *In situ* hybridization experiments were conducted to detect the expression of miR-3919 in the PCa tissue microarray. The sequence of the rhe miR-3919 probe was 5’-ACT GAG TCC TTT GTT CTC TGC-3’. Briefly, fresh and 5-μm thick paraffin sections were dewaxed and rehydrated. Antigen retrieval was performed using a citric acid solution in a pressure cooker. Then, the microarray was incubated with proteinase K (20 μg/mL) at 37°C for 20 min and washed in 0.5 M PBS for 5 min and three times. The microarray was then placed in a pre-hybridization solution for 2 h at 37°C and the probe was hybridized overnight at 37°C. The next day, the microarray was successively washed with 2 × SSC, 1 × SSC, and 0.5 × SSC (37°C, 10 min). The microarray was then incubated with signal probe hybridization solution overnight at 37°C and again washed with 2 × SSC, 1 × SSC, and 0.5 × SSC (37°C, 10 min). Cell nuclei were stained with DAPI (2 μg/mL), and the signals were visualized and imaged using a laser-scanning confocal microscope (Leica, Wetzlar, Germany).

### 2.12 Statistical analysis

Research data are presented as mean ± SD. Statistical analysis and graphing were performed using SPSS Statistics 23 or GraphPad Prism version 8.0.2. One-way ANOVA was used to analyze the differences between multiple groups, and the Student’s t-test was used to compare the differences between two groups. Statistical significance was set at *p <*0.05.

## 3 Results

### 3.1 Sulforaphane suppresses prostate cancer cell viability and DJ-1 expression

**Figure 1:**
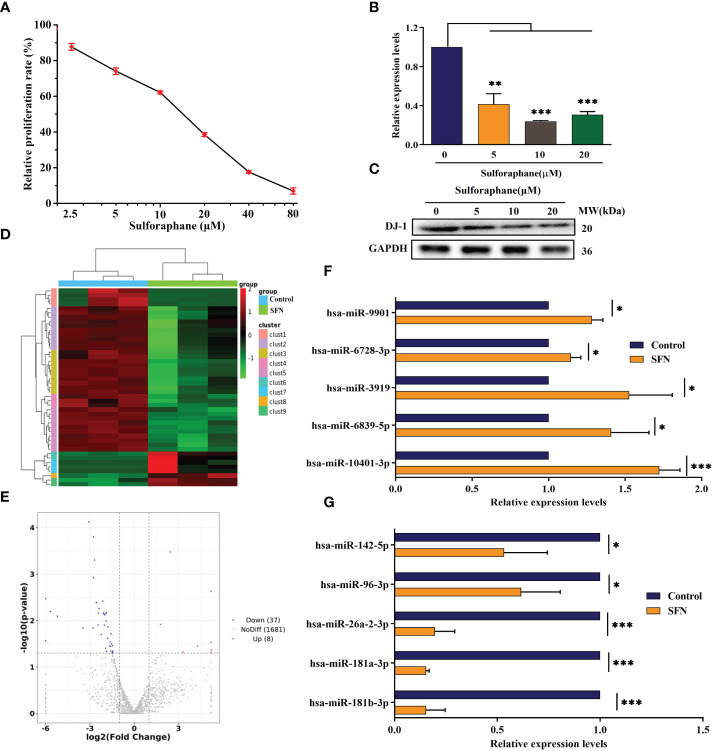
Effect of sulforaphane on cell viability and DJ-1 and miR-3919 expression in PC-3 cells. **(A)** CCK-8 assay was performed to determine cell viability. **(B)** qRT-PCR and **(C)** western blot were performed to detect DJ-1 mRNA and protein levels. **(D)** Heat map of miR-related gene expression of sulforaphane (10 μM) or DMSO-treated cells (FC >2, *P* <0.05). **(E)** Volcano plot analysis of the miR gene in the sulforaphane (10 μM) group compared with that in the DMSO group. **(F, G)** Five representative upregulated and five downregulated miRNAs in the small sequencing were verified by qRT-PCR (n = 3). Compared with control group, *p <0.05, **p <0.01, ***p <0.001; n = 3.

To confirm the effect of sulforaphane on the malignancy of PCa cells, a CCK-8 assay was performed. The result demonstrated that sulforaphane significantly decreased the viability of PC-3 cells in a dose-dependent manner (**Figure 1**). The viability of PC-3 cells after 48 h of exposure to 10 μM and 20 μM sulforaphane was reduced to 62.15% and 38.55%, respectively. The IC_50_ for PC-3 cells was 12.94 μM. Additionally, sulforaphane inhibited DJ-1 mRNA and protein expression in PC-3 cells. DJ-1, also known as *Park7*, was originally discovered as an oncogene that cooperates with ras to transform mouse NIH3T3 cells (22). Therefore, subsequent assays were conducted to investigate the regulatory effects of sulforaphane on DJ-1 expression.

Several studies have demonstrated that PC-3 cells are more sensitive to sulforaphane-mediated inhibition of cell viability than LNCaP cells (23–25). Therefore, we used the PC-3 and DU145 cell lines to explore the anti-tumor effects of miR-3919 or the combination of miR-3919 and sulforaphane in an *in vitro* study. Considering the literature reported data and the experimental results (10, 26, 27), cells treated with 10 μM sulforaphane for 48 h were used for the following assays.

Small RNA sequencing demonstrated that treatment with 10 μM sulforaphane upregulated eight miRNAs and downregulated 37 miRNAs (FC >2, *P* <0.05, **Figures 1D, E**). The top five upregulated and downregulated miRNAs were selected for quantitative RT-PCR assay to confirm the data obtained from small RNA sequencing. Prediction of miRNA target genes showed that DJ-1 was a functional target gene of miR-3919.

### 3.2 DJ-1 acted as a target for miR-3919 in PCa cells

**Figure 2:**
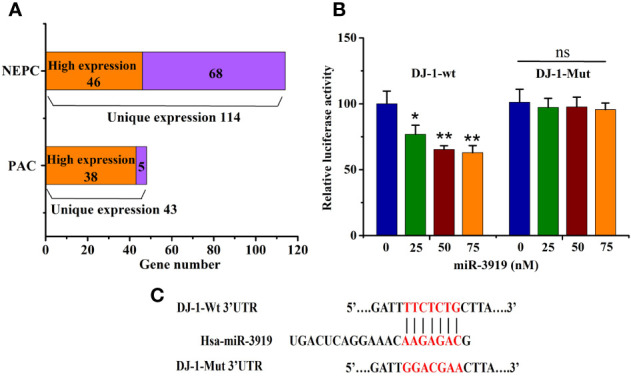
miR-3919 directly targets DJ-1. **(A)** Target Gene prediction analysis of miR-3919 expression in different PCa cell lines. **(B)** The dual-luciferase reporter assay was performed to detect the targeted binding of miR-3919 and DJ-1. **(C)** Schematic illustration of DJ-1 3’-UTR with putative binding sites for miR-3919. Compared to the NC group, *p<0.05, **p<0.01; n = 3.

Most PCa cases are characterized as prostatic adenocarcinoma (PAC) with luminal cell features and expression of AR and prostate-specific antigen (PSA) (28). Neuroendocrine prostate cancer (NEPC) is a highly malignant subtype of PCa that may arise *de novo* or emerge in patients (29). To further investigate the effects of miR-3919 on PCa, target gene analysis of miR-3919 between two PAC cell lines (LNCaP and 22RV1) and two NEPC cell lines (PC-3 and DU145) was performed using the miRDB database. As shown in **Figure 2A**, the results demonstrated that 43 of the predicted target genes were expressed only in the PAC cell lines and 114 were expressed only in the NEPC cell lines. The levels of genes expressed in both cell types were compared using methods reported in the literature (30); 38 target genes were highly expressed in PAC cell lines and 46 were highly expressed in NEPC cell lines. Taken together, miR-3919 regulates more target genes in NEPC cell lines than PAC cell lines, which may indicate that miR-3919 may play a greater role in NEPC cell lines.

**Figure 3:**
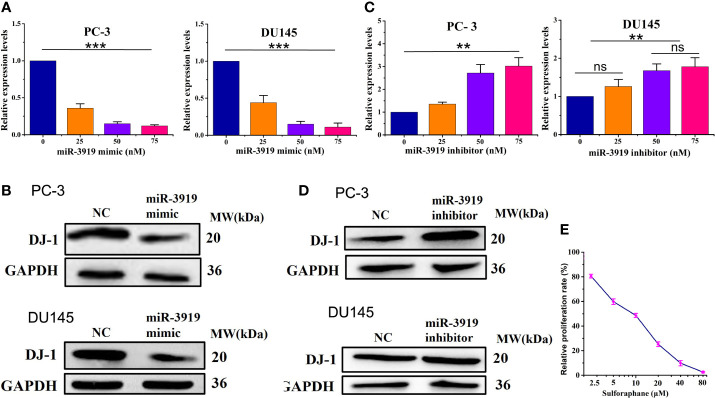
miR-3919 suppresses DJ-1 expression in PCa cells. **(A, B)** qRT-PCR and western blotting were performed to detect the effects of miR-3919 mimic on DJ-1 mRNA and protein levels. **(C, D)** qRT-PCR and western blotting were performed to detect the effects of miR-3919 inhibitor on DJ-1 mRNA and protein levels. Compared with NC group, ns: no significance; **p <0.01, ***p <0.001; n = 3. **(E)** CCK-8 assays were conducted to detect the viability of PC-3 cells after SFN and miR-3919 mimic treatment.

The puative binding site (**Figure 2C**) between miR-3919 and DJ-1 was predicted using TargetScan database. The results of the luciferase-based reporter assay (**Figure 2B**) demonstrated that miR-3919 mimics significantly reduced relative luciferase activity in the DJ-1-Wt 3’UTR group, but had no effect on that of DJ-1-Mut 3’-UTR compared with NC miRNA in PC-3 cells. Furthermore, the miR-3919 mimic or NC was transfected into the PC-3 and DU145 cells. The results of qRT-PCR and western blotting indicated that the expression of DJ-1 was significantly downregulated when miR-3919 was overexpressed (**Figures 3A, B**). The miR-3919 inhibitor significantly increased DJ-1 mRNA and protein expression in PC-3 and DU145 cells (**Figures 3C, D**). Therefore, the results showed that DJ-1 is a direct target gene of miR-3919 and is negatively regulated by miR-3919 in PCa cells.

As shown in **Figures 2B**, **Figure 3A, C**, the effects caused by the 50 nM dose were similar and much better than that of 25 nM. Therefore, we chose 50 nM for subsequent experiments. We used SFN in miR-3919 mimic (50 nM) transfected PC-3 and got a IC_50_ of SFN of 8.36 μM (**Figure 3E**).

### 3.3 miR-3919 inhibited the proliferation, migration, and invasion of PCa cells, and promoted apoptosis

**Figure 4:**
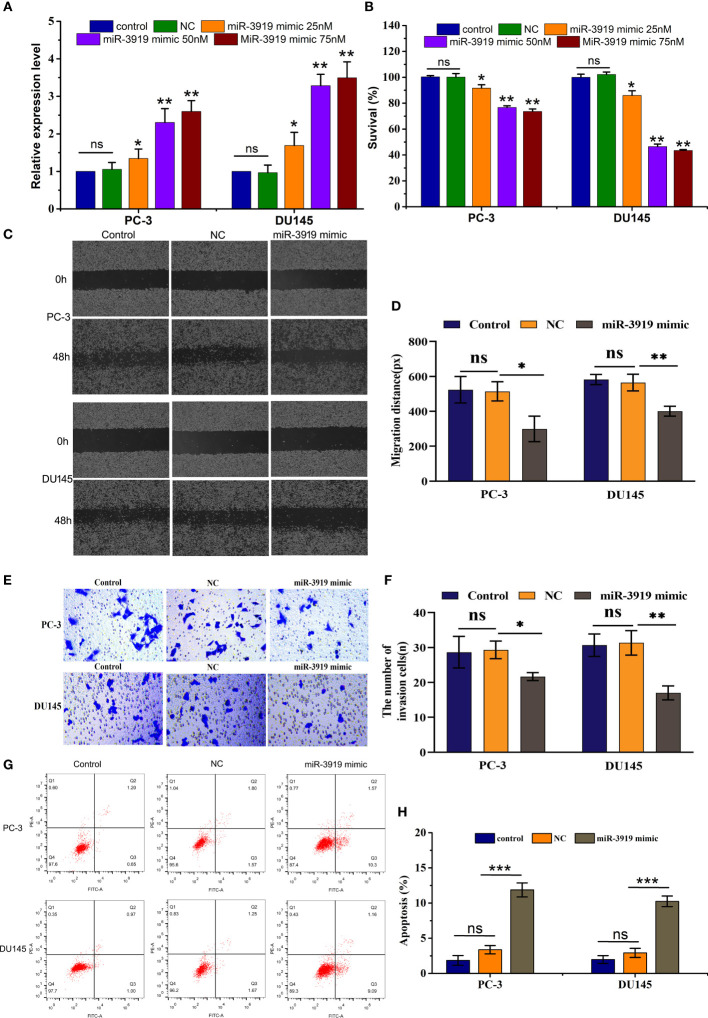
miR-3919 suppresses the malignancy of PCa cells. **(A)** qRT-PCR was performed to measure miR-3919 expression in PC-3 and DU145 cells transfected with the NC or miR-3919 mimic. **(B)** CCK-8 assays were conducted to detect cell viability in PC-3 and DU145 cells transfected with the NC or miR-3919 mimic. **(C, D)** Cell migration distance was determined in PC-3 and DU145 cells transfected with NC or miR-3919 mimic. **(E, F)** Cell invasion levels were examined in PC-3 and DU145 cells transfected with the NC or miR-3919 mimic. **(G, H)** Apoptosis was detected in PC-3 and DU145 cells transfected with NC or miR-3919 mimic. Compared with NC group, ns: no significance; *p < 0.05, **p < 0.01, ***p < 0.001; n = 3.

To further elucidate the role of miR-3919 in PCa cells, we performed functional studies in PC-3 and DU145 cells. First, miR-3919 mimic or NC was transfected into PC-3 and DU145 cells. The results showed that miR-3919 expression was upregulated by over 2-fold (**Figure 4A**). As shown in **Figure 4B**, the miR-3919 mimic significantly reduced cell viability compared to the NC group. Wound healing and transwell assays also demonstrated that the miR-3919 mimic significantly inhibited cell migration and invasion (**Figures 4C–F**). Furthermore, flow cytometry was performed, and the results indicated that miR-3919 mimics promoted apoptosis (**Figures 4G, H**).

### 3.4 miR-3919 agomir inhibited tumor growth *in vivo*

**Figure 5:**
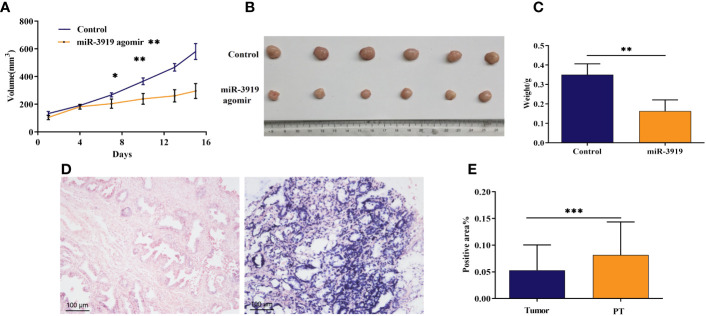
The miR-3919 agomir inhibited tumor growth *in vivo*, and miR‐3919 showed low expression in PCa tissues. **(A)** Tumor volume of xenografts from the control and miR-3919 agomir groups was monitored (n = 6). **(B)** Xenograft tumors isolated from control and miR-3919 agomir groups were photographed on day 15 (n = 6). **(C)** Xenograft tumors isolated from control and miR-3919 agomir groups were weighed (n = 6). **(D)** Representative results of miR-3919 *in situ* hybridization in a PCa tissue microarray (n = 40). **(E)** Statistical analysis of miR-3919 *in situ* hybridization in a PCa tissue microarray (n = 40). Compared with control group, *p < 0.05, **p < 0.01, ***p < 0.001.

To further explore the role of miR-3919 in PCa tumorigesis, we established an *in vivo* xenograft model. As indicated in **Figure 5A**, miR-3919 agomir significantly decreased tumor volume (*p* <0.01). Reduced tumor weight was also observed in the miR-3919 agomir group (0.16 ± 0.06 g) than in the control group (0.35 ± 0.06 g, *p* <0.01, **Figures 5B, C**). These results indicate that miR-3919 inhibits tumor growth *in vivo*. Subsequently, an *in situ* hybridization assay performed in PCa tissue microarray validated that miR-3919 expression in PCa tissues was lower than that in peritumoral tissues (**Figures 5D, E**), which also demonstrated that miR-3919 exerted antitumor effects.

### 3.5 Sulforaphane inhibited the malignancy of PCa cells partly by regulating miR-3919/DJ-1 pathway

**Figure 6:**
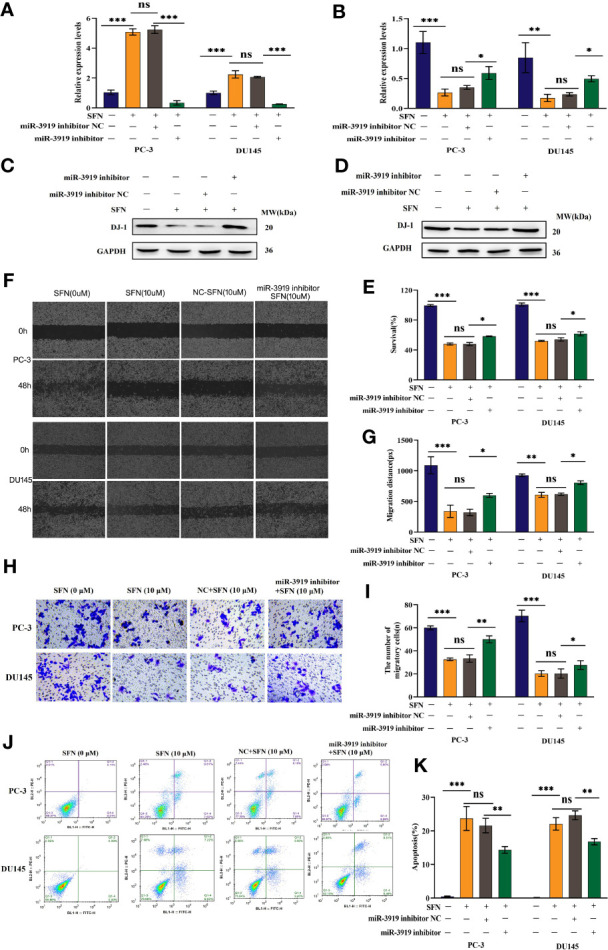
Sulforaphane inhibited the malignancy of PCa cells via miR-3919. **(A, B)** After transfection with miR-3919 inhibitor and sulforaphane treatment, qRT-PCR was performed to detect miR-3919 and DJ-1 expression in PCa cells. **(C, D)** After transfection with miR-3919 inhibitor and sulforaphane treatment, western blotting was performed to detect DJ-1 expression in PC-3 and DU145 cells, respectively. **(E)** After transfection with miR-3919 inhibitor and sulforaphane treatment, the CCK-8 assay was performed in PCa cells. **(F, G)** After transfection with miR-3919 inhibitor and sulforaphane treatment, cell migration distance was determined in PCa cells. **(H, I)** After transfection with miR-3919 inhibitor and sulforaphane treatment, cell invasion levels were examined in PCa cells. **(J, K)** Apoptosis was detected in PCa cells after transfection with the miR-3919 inhibitor and sulforaphane treatment. ns: no significance; *p < 0.05, **p < 0.01, ***p < 0.001; n = 3.

Based on the above results, we investigated whether sulforaphane regulates the malignancy of PCa cells via the miR-3919/DJ-1 pathway. First, PCa cells were treated with 10 μM sulforaphane for 48 h and transfected with miR-3919 inhibitor. As shown in **Figures 6A, B**, the miR-3919 inhibitor significantly reversed the effects of sulforaphane on the mRNA expression of miR-3919 and DJ-1 in PC-3 and DU145 cells. The miR-3919 inhibitor also notably attenuated the inhibitory effect of sulforaphane on the protein expression of DJ-1 in both PCa cell lines (**Figures 6C, D**). The results of CCK-8, wound healing, and transwell assays demonstrated that sulforaphane significantly repressed PCa cell proliferation, migration, and invasion, whereas the miR-3919 inhibitor partially reversed these effects (**Figures 6E–I**). The miR-3919 inhibitor also partly reversed the effects of sulforaphane on the apoptosis of PCa cells (**Figures 6J, K**). These results indicate that the miR-3919/DJ-1 axis is a significant downstream effector of sulforaphane.

## 4 Discussion

Here, we report the expression profile of miRNAs in SFN-treated PCa cells for the first time. Among validated miRNAs whose expression was upregulated in SFN-treated PC-3 cells, miR-181b-3p was previously reported to promote epithelial–mesenchymal transition in breast cancer cells (20), and the occurrence and development of colorectal cancer (31). miR-142-5p functions as an oncogene in PCa (32), and its expression level is negatively correlated with the overall survival of patients with PCa (33). Interestingly, SFN suppressed miR-142-5p expression in the PC-3 cells. Taken together, these results suggest that the aberrant expression of miR-181b-3p and miR-142-5p plays an important role in the suppressive effect of SFN on the growth of PC-3 cells.

Importantly, we not only detected that miR-3919 expression was significantly increased in SFN-treated PC-3 cells, but also found that miR-3919 has a tumor-suppressive role both *in vitro* and *in vivo*. Consistent with these results, our tissue microarray analysis revealed that miR-3919 expression is lower in PCa specimens than in adjacent normal tissues. Therefore, it can be concluded that miR-3919 acts as a tumor suppressor in PCa.

Our results demonstrate that the tumor-suppressive effect of SFN depends on its targeting of miR-3919 expression, given that it was significantly reduced in PC-3 cells transfected with the miR-3919 inhibitor. In the present study, we validated that DJ-1 is a target of miR-3919. DJ-1, a protein with multiple functions, was originally identified as a novel oncogene that exhibits significant transforming activity in cooperation with c-Myc or H-Ras (20). It has been reported to be overexpressed and is related to poor prognosis in many types of cancers (34). It not only acts as a repressor of the tumor suppressor PTEN (34–36) but also has gene transcription-regulating functions via its interaction with distinct transcriptional factors (37). Interestingly, DJ-1 binds androgen receptors directly in LNCaP cells and mediates its activity, probably for the progression of prostate cancer to androgen independence (38). One possible mechanism is that DJ-1 promotes PCa growth through autophagy inhibition in PCa cells (39). Considering that knockdown of DJ-1 expression reduces the growth of PC-3 cells (40), it is speculated that miR-3919 inhibits the growth of PCa in a way depending on targeting of DJ-1. As supporting evidence, we found that SFN increased the expression of miR-3919 but reduced the level of DJ-1 protein. However, the inhibitory effects of SPN on cell growth and DJ-1 expression were significantly downregulated in the presence of miR-3919 inhibitor. To prove that the miR-3919/DJ-1 axis is important for tumor-suppressive function of SFN, it should be evaluated whether ectopic expression of DJ-1 can reduce the efficiency of the inhibitory effect of SFN on the growth rate of PCa cells.

**Figure 7:**
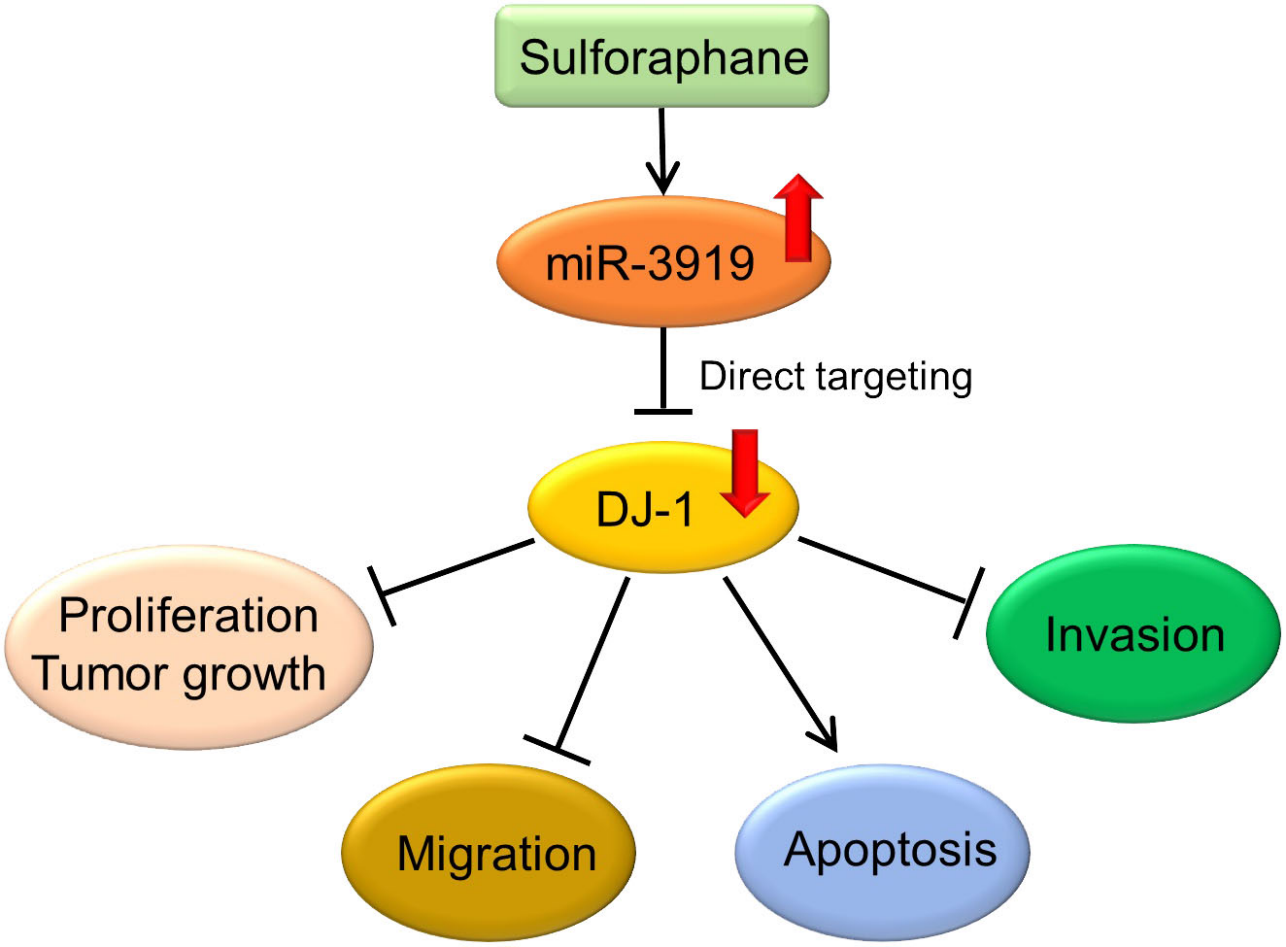
Sulforaphane inhibits PCa cells through the miR-3919/DJ-1 axis.

In conclusion, we found that sulforaphane repressed the malignancy of PCa cells through the miR-3919/DJ-1 pathway (**Figure 7**) and miR-3919 was found to be a tumor suppressor in PCa in the present study. To the best of our knowledge, the function of miR-3919 has not been reported previously. Upregulation of miR-3919 has been shown to be important for inhibitory effect of SFN on growth rate of PCa cells. Therefore, targeting miR-3919 may be an extra way to develop new drug therapies for PCa, especially for NEPC, given that the PC-3 cell line represents an NEPC cell line without endogenous AR expression (41).

## Data availability statement

The raw data supporting the conclusions of this article will be made available by the authors, without undue reservation.

## Ethics statement

The animal study protocol was approved by the Animal Ethics Committee of the Shanghai Institute for Biomedical and Pharmaceutical Technologies. The study was conducted in accordance with local legislation and institutional requirements.

## Author contributions

FZ: Data curation, Investigation, Methodology, Writing – original draft. XW: Data curation, Methodology, Writing – original draft. JZ: Formal analysis, Methodology, Writing – original draft. MS: Data curation, Funding acquisition, Visualization, Writing – review & editing. RL: Data curation, Funding acquisition, Project administration, Supervision, Writing – review & editing.

## References

[1] Ghosh S Hazra J Pal K Nelson VK Pal M. Prostate cancer: Therapeutic prospect with herbal medicine. Curr Res Pharmacol Drug Discovery. (2021) 2:100034. doi: 10.1016/j.crphar.2021.100034 PMC8663990 34909665

[2] Termini D Den Hartogh DJ Jaglanian A Tsiani E. Curcumin against prostate cancer: Current evidence. Biomolecules. (2020) 10(11):1536. doi: 10.3390/biom10111536 33182828 PMC7696488

[3] Hahn AW Siddiqui BA Leo J Dondossola E Basham KJ Miranti CK. Cancer cell-extrinsic roles for the androgen receptor in prostate cancer. Endocrinology. (2023) 164(6): bqad078. doi: 10.1210/endocr/bqad078 37192413 PMC10413433

[4] Arrighetti N Beretta GL. miRNAs as therapeutic tools and biomarkers for prostate cancer. Pharmaceutics. (2021) 13(3):380. doi: 10.3390/pharmaceutics13030380 33805590 PMC7999286

[5] Rutz J Thaler S Maxeiner S Chun FK Blaheta RA. Sulforaphane reduces prostate cancer cell growth and proliferation in vitro by modulating the cdk-cyclin axis and expression of the CD44 variants 4, 5, and 7. Int J Mol Sci. (2020) 21(22):8274. doi: 10.3390/ijms21228724 33218199 PMC7699211

[6] Hammersen F Pursche T Fischer D Katalinic A Waldmann A. Use of complementary and alternative medicine among young patients with breast cancer. Breast Care (Basel). (2020) 15:163–70. doi: 10.1159/000501193 PMC7204767 32398985

[7] Naujokat C McKee DL. The "Big five" Phytochemicals targeting cancer stem cells: Curcumin, EGCG, sulforaphane, resveratrol and genistein. Curr Med Chem. (2021) 28:4321–42. doi: 10.2174/0929867327666200228110738 32107991

[8] Dacosta C Bao Y. The role of microRNAs in the chemopreventive activity of sulforaphane from cruciferous vegetables. Nutrients. (2017) 9(8):902. doi: 10.3390/nu9080902 28825609 PMC5579695

[9] Ramos-Gomez M Kwak MK Dolan PM Itoh K Yamamoto M Talalay P. Sensitivity to carcinogenesis is increased and chemoprotective efficacy of enzyme inducers is lost in nrf2 transcription factor-deficient mice. Proc Natl Acad Sci U.S.A. (2001) 98:3410–5. doi: 10.1073/pnas.051618798 PMC30667 11248092

[10] Kim SH Singh SV. D,L-Sulforaphane causes transcriptional repression of androgen receptor in human prostate cancer cells. Mol Cancer Ther. (2009) 8:1946–54. doi: 10.1158/1535-7163.MCT-09-0104 PMC2779717 19584240

[11] Traka MH Melchini A Mithen RF. Sulforaphane and prostate cancer interception. Drug Discovery Today. (2014) 19:1488–92. doi: 10.1016/j.drudis.2014.07.007 25051139

[12] Rajendran P Kidane AI Yu TW Dashwood WM Bisson WH Lohr CV. HDAC turnover, CtIP acetylation and dysregulated DNA damage signaling in colon cancer cells treated with sulforaphane and related dietary isothiocyanates. Epigenetics. (2013) 8:612–23. doi: 10.4161/epi.24710 PMC3857341 23770684

[13] Zhang C Su ZY Khor TO Shu L Kong AN. Sulforaphane enhances Nrf2 expression in prostate cancer TRAMP C1 cells through epigenetic regulation. Biochem Pharmacol. (2013) 85:1398–404. doi: 10.1016/j.bcp.2013.02.010 PMC4123317 23416117

[14] Dinkova-Kostova AT Fahey JW Kostov RV Kensler TW. KEAP1 and done? Targeting the NRF2 pathway with sulforaphane. Trends Food Sci Technol. (2017) 69:257–69. doi: 10.1016/j.tifs.2017.02.002 PMC5725197 29242678

[15] Yang F Wang F Liu Y Wang S Li X Huang Y. Sulforaphane induces autophagy by inhibition of HDAC6-mediated PTEN activation in triple negative breast cancer cells. Life Sci. (2018) 213:149–57. doi: 10.1016/j.lfs.2018.10.034 30352240

[16] Hu M Zheng Y Liao J Wen L Cheng J Huang J. miR21 modulates the Hippo signaling pathway via interference with PP2A Bbeta to inhibit trophoblast invasion and cause preeclampsia. Mol Ther Nucleic Acids. (2022) 30:143–61. doi: 10.1016/j.omtn.2022.09.006 PMC9547189 36250210

[17] Peng Y Croce CM. The role of MicroRNAs in human cancer. Signal Transduct Target Ther. (2016) 1:15004. doi: 10.1038/sigtrans.2015.4 29263891 PMC5661652

[18] Menon A Abd-Aziz N Khalid K Poh CL Naidu R. miRNA: A promising therapeutic target in cancer. Int J Mol Sci. (2022) 23. doi: 10.3390/ijms231911502 PMC9569513 36232799

[19] Hosseinahli N Aghapour M Duijf PHG Baradaran B. Treating cancer with microRNA replacement therapy: A literature review. J Cell Physiol. (2018) 233:5574–88. doi: 10.1002/jcp.26514 29521426

[20] Yoo JO Kwak SY An HJ Bae IH Park MJ Han YH. miR-181b-3p promotes epithelial-mesenchymal transition in breast cancer cells through Snail stabilization by directly targeting YWHAG. Biochim Biophys Acta. (2016) 1863:1601–11. doi: 10.1016/j.bbamcr.2016.04.016 27102539

[21] Wang C Li W Hu Q Feng N Liu C Shi N. Transgenic construction and functional miRNA analysis identify the role of miR-7 in prostate cancer suppression. Oncogene. (2022) 41:4645–57. doi: 10.1038/s41388-022-02461-0 36088503

[22] Nagakubo D Taira T Kitaura H Ikeda M Tamai K Iguchi-Ariga SM. DJ-1, a novel oncogene which transforms mouse NIH3T3 cells in cooperation with ras. Biochem Biophys Res Commun. (1997) 231:509–13. doi: 10.1006/bbrc.1997.6132 9070310

[23] Guo J Ma K Xia HM Chen QK Li L Deng J. Androgen receptor reverts dexamethasone−induced inhibition of prostate cancer cell proliferation and migration. Mol Med Rep. (2018) 17:5887–93. doi: 10.3892/mmr PMC5866034 29436611

[24] Fiandalo MV Wilton JH Mantione KM Wrzosek C Attwood KM Wu Y. Serum-free complete medium, an alternative medium to mimic androgen deprivation in human prostate cancer cell line models. Prostate. (2018) 78:213–21. doi: 10.1002/pros.23459 PMC5768451 29194687

[25] Liang ZR Qu LH Ma LM. Differential impacts of charcoal-stripped fetal bovine serum on c-Myc among distinct subtypes of breast cancer cell lines. Biochem Biophys Res Commun. (2020) 526:267–72. doi: 10.1016/j.bbrc.2020.03.049 32209261

[26] Pei Y Wu B Cao Q Wu L Yang G. Hydrogen sulfide mediates the anti-survival effect of sulforaphane on human prostate cancer cells. Toxicol Appl Pharmacol. (2011) 257:420–8. doi: 10.1016/j.taap.2011.09.026 22005276

[27] Myzak MC Hardin K Wang R Dashwood RH Ho E. Sulforaphane inhibits histone deacetylase activity in BPH-1, LnCaP and PC-3 prostate epithelial cells. Carcinogenesis. (2006) 27:811–9. doi: 10.1093/carcin/bgi265 PMC2276576 16280330

[28] Shen MM Abate-Shen C. Molecular genetics of prostate cancer: new prospects for old challenges. Genes Dev. (2010) 24:1967–2000. doi: 10.1101/gad.1965810 20844012 PMC2939361

[29] Merkens L Sailer V Lessel D Janzen E Greimeier S Kirfel J. Aggressive variants of prostate cancer: underlying mechanisms of neuroendocrine transdifferentiation. J Exp Clin Cancer Res. (2022) 41:46. doi: 10.1186/s13046-022-02255-y 35109899 PMC8808994

[30] Wang L Feng Z Wang X Wang X Zhang X. DEGseq: an R package for identifying differentially expressed genes from RNA-seq data. Bioinformatics. (2010) 26:136–8. doi: 10.1093/bioinformatics/btp612 19855105

[31] Jiang Y Qiu Q Jing X Song Z Zhang Y Wang C. Cancer-associated fibroblast-derived exosome miR-181b-3p promotes the occurrence and development of colorectal cancer by regulating SNX2 expression. Biochem Biophys Res Commun. (2023) 641:177–85. doi: 10.1016/j.bbrc.2022.12.026 36535076

[32] Tan Y Chen ZY Wang L Wang M Liu XH. MiR-142-3p functions as an oncogene in prostate cancer by targeting FOXO1. J Cancer. (2020) 11:1614–24. doi: 10.7150/jca.41888 PMC6995382 32047567

[33] Zhou Z Wu X Zhou Y Yan W. Long non-coding RNA ADAMTS9-AS1 inhibits the progression of prostate cancer by modulating the miR-142-5p/CCND1 axis. J Gene Med. (2021) 23:e3331. doi: 10.1002/jgm.3331 33704879

[34] Cao J Chen X Ying M He Q Yang B. DJ-1 as a therapeutic target against cancer. Adv Exp Med Biol. (2017) 1037:203–22. doi: 10.1007/978-981-10-6583-5_13 29147911

[35] Raymond HK Peters M Jang Y Shi W Pintilie M Fletcher GC. DJ-1, a novel regulator of the tumor suppressor PTEN. Cancer Cell. (2005) 7:263–73. doi: 10.1016/j.ccr.2005.02.010 15766664

[36] Lee YJ Kim WI Park TH Bae JH Nam HS Cho SW. Upregulation of DJ-1 expression in melanoma regulates PTEN/AKT pathway for cell survival and migration. Arch Dermatol Res. (2020) 313(7):583-91. doi: 10.1007/s00403-020-02139-1 32959108

[37] Takahashi-Niki K Niki T Iguchi-Ariga SMM Ariga H. Transcriptional regulation of DJ-1. Adv Exp Med Biol. (2017) 1037:89–95. doi: 10.1007/978-981-10-6583-5_7 29147905

[38] Tillman JE Lu A Ke M Zhu W Ye X Wang G. DJ-1 binds androgen receptor directly and mediates its activity in hormonally treated prostate cancer cells. Cancer Res. (2007) 67:4630–7. doi: 10.1158/0008-5472.CAN-06-4556 17510388

[39] Qin X. DJ-1 inhibits autophagy activity of prostate cancer cells by repressing JNK-Bcl2-Beclin1 signaling. Cell Biol Int. (2020) 44:937–46. doi: 10.1002/cbin.11290 31868268

[40] Hod Y. Differential control of apoptosis by DJ-1 in prostate benign and cancer cells. J Cell Biochem. (2004) 92:1221–33. doi: 10.1002/jcb.20159 15258905

[41] Tai S Sun Y Squires JM Zhang H Oh WK Liang CZ. PC3 is a cell line characteristic of prostatic small cell carcinoma. Prostate. (2011) 71:1668–79. doi: 10.1002/pros.21383 PMC3426349 21432867

