# Supplementary material for: Sulforaphane inhibits the growth of prostate cancer by regulating the microRNA-3919/DJ-1 axis
Source: Front Oncol. 2024 Mar 7;14:1361152. doi: 10.3389/fonc.2024.1361152 (PMC10955061; doi:10.3389/fonc.2024.1361152)
Supplement: Supplementary file 1 [file DataSheet_1.pdf]

Uncropped western blots - not for publication

Fig.1 C

1 2 3 4

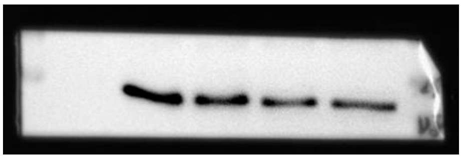

DJ-1 for Fig.1 C

1 SFN (0 µM);

2 SFN (5 µM);

3 SFN (10 µM);

4 SFN (20 µM);

1 2 3 4

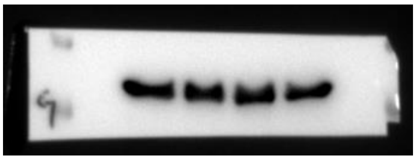

GAPDH for Fig.1 C

1 SFN (0 µM);

2 SFN (5 µM)

3 SFN (10 µM)

4 SFN (20 µM)

Lane 1, 2, 3 and 4 for Fig.1 C

Fig.3 B

1 2 3 **4** **5** 6 7 8 9

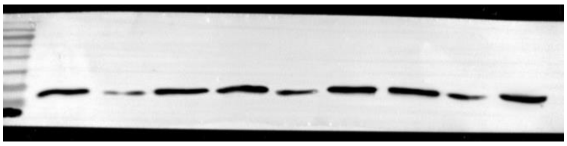

PC-3 cells DJ-1 for Fig.3 B

1 DMSO

2 Compound 171

3 DMSO

4 NC

5 miR-3919 mimic

6 DMSO

7 Compound 172

8 Compound 173

9 Compound 174

1 2 3 **4** **5** 6 7 8 9

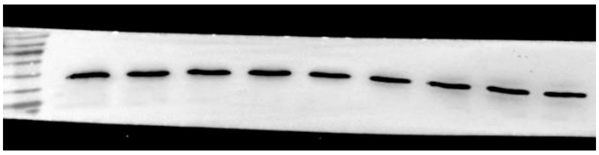

PC-3 cells GAPDH for Fig.3 B

1 DMSO

2 Compound 171

3 DMSO

4 NC

5 miR-3919 mimic

6 DMSO

7 Compound 172

7 Compound 173

7 Compound 174

Lane 4 and 5 for Fig.3 B

1 2 3 **4** **5** 6 7 8 9

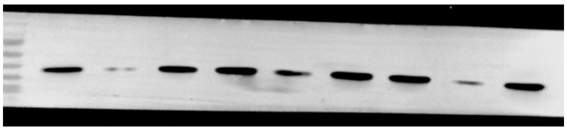

DU145 cells DJ-1 for Fig.3 B

1 DMSO

2 Compound 171

3 DMSO

4 NC

5 miR-3919 mimic

6 DMSO

7 Compound 172

8 Compound 173

9 Compound 174

1 2 3 **4** **5** 6 7 8 9

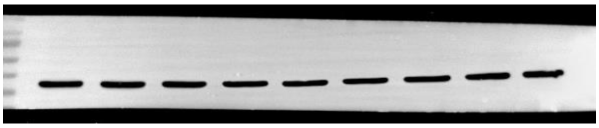

DU145 cells GAPDH for Fig.3 B

1 DMSO

2 Compound 171

3 DMSO

4 NC

5 miR-3919 mimic

6 DMSO

7 Compound 172

7 Compound 173

7 Compound 174

Lane 4 and 5 for Fig.3 B

Fig.3 D and Fig.6 C

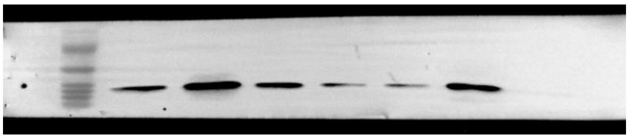

PC-3 cells DJ-1 for Fig.3 D and Fig.6 C

1 NC

2 miR-3919 inhibitor

3 SFN (0 μM)

4 SFN (10 μM)

5 NC+ SFN (10 μM)

6 miR-3919 inhibitor+ SFN (10 μM)

Lane 1 and 2 for Fig.3 D

Lane 3, 4, 5 and 6 for Fig.6 C

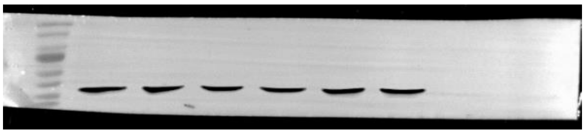

PC-3 cells GAPDH for Fig.3 D and Fig.6 C

1 NC

2 miR-3919 inhibitor

3 SFN (0 μM)

4 SFN (10 μM)

5 NC+ SFN (10 μM)

6 miR-3919 inhibitor+ SFN (10 μM)

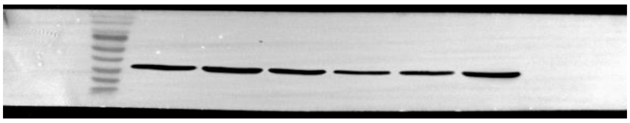

DU145 cells DJ-1 for Fig.3 D and Fig.6 C

1 NC

2 miR-3919 inhibitor

3 SFN (0 μM)

4 SFN (10 μM)

5 NC+ SFN (10 μM)

6 miR-3919 inhibitor+ SFN (10 μM)

Lane 1 and 2 for Fig.3 D

Lane 3, 4, 5 and 6 for Fig.6 C

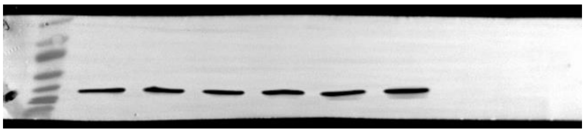

DU145 cells GAPDH for Fig.3 D and Fig.6 C

1 NC

2 miR-3919 inhibitor

3 SFN (0 μM)

4 SFN (10 μM)

5 NC+ SFN (10 μM)

6 miR-3919 inhibitor+ SFN (10 μM)

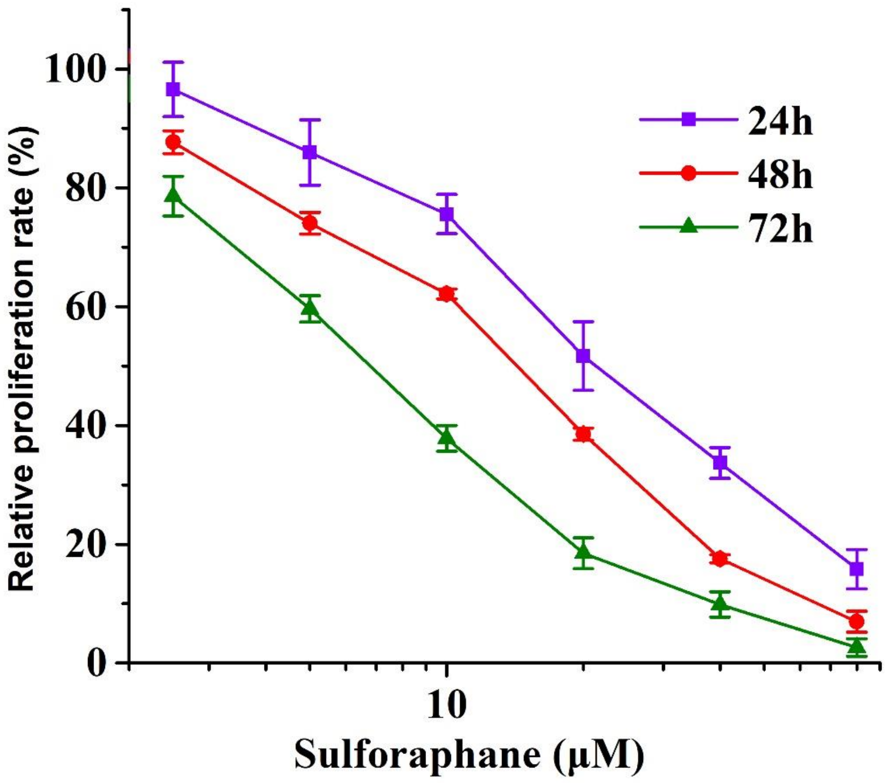

Fig. S1 Relative proliferation rate of PC-3 cells after treated with SFN for 24, 48, 72h.
